# Identification of asthma-related genes using asthmatic blood eQTLs of Korean patients

**DOI:** 10.1186/s12920-023-01677-7

**Published:** 2023-10-24

**Authors:** Dong Jun Kim, Ji Eun Lim, Hae-Un Jung, Ju Yeon Chung, Eun Ju Baek, Hyein Jung, Shin Young Kwon, Han Kyul Kim, Ji-One Kang, Kyungtaek Park, Sungho Won, Tae-Bum Kim, Bermseok Oh

**Affiliations:** 1 Department of Biochemistry and Molecular Biology, School of Medicine, Kyung Hee University, Seoul, Republic of Korea; 2 Department of Biomedical Science, Graduate School, Kyung Hee University, Seoul, Korea; 3 Mendel Inc, Seoul, Republic of Korea; 4 Interdisciplinary Program of Bioinformatics, Seoul National University, Seoul, Republic of Korea; 5 Department of Public Health Sciences, School of Public Health, Seoul National University, Seoul, Republic of Korea; 6 Department of Allergy and Clinical Immunology, Asan Medical Center, University of Ulsan College of Medicine, Seoul, Republic of Korea

**Keywords:** Asthma, Expression quantitative trait loci, Genome-wide association study, Colocalization, Summary-based Mendelian Randomization, Transcriptome-wide association study

## Abstract

**Background:**

More than 200 asthma-associated genetic variants have been identified in genome-wide association studies (GWASs). Expression quantitative trait loci (eQTL) data resources can help identify causal genes of the GWAS signals, but it can be difficult to find an eQTL that reflects the disease state because most eQTL data are obtained from normal healthy subjects.

**Methods:**

We performed a blood eQTL analysis using transcriptomic and genotypic data from 433 Korean asthma patients. To identify asthma-related genes, we carried out colocalization, Summary-based Mendelian Randomization (SMR) analysis, and Transcriptome-Wide Association Study (TWAS) using the results of asthma GWASs and eQTL data. In addition, we compared the results of disease eQTL data and asthma-related genes with two normal blood eQTL data from Genotype-Tissue Expression (GTEx) project and a Japanese study.

**Results:**

We identified 340,274 cis-eQTL and 2,875 eGenes from asthmatic eQTL analysis. We compared the disease eQTL results with GTEx and a Japanese study and found that 64.1% of the 2,875 eGenes overlapped with the GTEx eGenes and 39.0% with the Japanese eGenes. Following the integrated analysis of the asthmatic eQTL data with asthma GWASs, using colocalization and SMR methods, we identified 15 asthma-related genes specific to the Korean asthmatic eQTL data.

**Conclusions:**

We provided Korean asthmatic cis-eQTL data and identified asthma-related genes by integrating them with GWAS data. In addition, we suggested these asthma-related genes as therapeutic targets for asthma. We envisage that our findings will contribute to understanding the etiological mechanisms of asthma and provide novel therapeutic targets.

**Supplementary Information:**

The online version contains supplementary material available at 10.1186/s12920-023-01677-7.

## Background

Asthma is one of the most common chronic respiratory diseases worldwide, which affected 262 million people in 2019 [1] and is a complex disease with multiple endotypes and different underlying pathological mechanisms [2]. Both genetic and environmental factors contribute to asthma development, and the heritability of asthma is 35–95% [3]. Large population-based genome-wide association studies (GWASs) have identified more than 200 lead single-nucleotide polymorphisms (SNPs) associated with asthma or asthma subtypes [4–10]. However, most asthma GWAS SNPs are located in non-coding regions; therefore, it is unclear which genes are causative, leading to a lack of understanding of the biological pathways involved in asthma.

Expression quantitative trait loci (eQTL) are genetic variants that affect the expression of genes and are discovered via association analyses between genetic variants and gene expression levels [11]. eQTL data have been used to identify causal genes from the associated SNPs of GWASs by linking the genetic variant to changes in the gene expression, which consequently helps to understand the underlying biological pathways [12–14]. To better serve this, several statistical methods have been developed to integrate GWAS results with eQTL data. COLOC is a statistical framework that estimates the probability of sharing the same causal genetic variants in GWASs and eQTL [15]. Summary-based Mendelian Randomization (SMR) is another method used to identify the causative genes from GWAS results by assessing the association between the variations in gene expressions and a trait; SMR is free from the confounding of non-genetic factors [16]. In addition, the transcriptome-wide association study (TWAS) is a statistical approach for identifying candidate causal genes using the results of GWASs and eQTL data [17]. In the TWAS analysis, gene expression levels are imputed by eQTLs for a gene, and the association between the imputed gene expression levels and a trait is tested to estimate the statistical significance of each gene-disease association. Recent studies have further integrated GWAS results with eQTL, protein quantitative trait loci (pQTL), and epigenetic data to identify target genes [18, 19].

The eQTL data resources for various human tissues have increased [20, 21]; however, most eQTL resources are constructed from healthy human subjects. The regulation of gene expression has been shown to be context-specific; therefore, gene expression may differ depending on disease states, as well as tissue and cell types [22, 23]. Large eQTL studies across various human tissues have been conducted by the Genotype-Tissue Expression (GTEx) consortium [20], and tissue- or cell-type eQTL databases have been analyzed to identify the causative genes from GWAS results [23, 24]. A few disease-specific eQTL databases have been constructed, however, the usefulness of these has not been evaluated well [25–27]. Certain eQTL effects may be influenced by disease status, therefore this eQTL would not be identified by the eQTL data constructed from healthy subjects.

In the present study, we performed an eQTL analysis using RNA-seq data of peripheral blood mononuclear cells (PBMCs) from 433 Korean patients with asthma, as well as their genotype data. We compared the results of the asthmatic eQTL analysis with two healthy blood eQTL data and found not only substantial overlaps but also unique eQTLs in the asthma patients. Furthermore, we integrated the asthma eQTLs with two asthma GWAS summaries and identified asthma susceptibility genes related to Korean asthmatic eQTL data. These asthma eQTL data can be used to further identify asthma susceptibility genes from asthma GWASs, resulting in enhanced knowledge regarding the regulatory pathways and biological processes of asthma.

## Methods

### Study subjects

A total of 436 Korean patients with asthma were obtained from the Allergy Asthma Center of the Asan Medical Center, Seoul, Republic of Korea between January 2005 and December 2018. The inclusion criteria were as follows: 1) symptoms such as dyspnea, wheezing or cough for more than three months; and 2) airway hyperresponsiveness, as indicated by a 20% reduction in forced expiratory volume in 1 s (FEV1) with a methacholine dose of 16 mg/mL [provocation concentration (PC) causing a 20% fall in FEV1; PC_20_] through a provocation test or airway reversibility, as indicated by an increase in FEV1 of > 12% (and at least 200 mL) following the inhalation of a short-acting β-agonist. Patients with severe lung damage, bronchiectasis or a history of lung resection were excluded.

Blood samples from each subject were collected for the genotyping and transcriptomic analysis. PBMCs were isolated from whole blood samples (2 tubes of 10 ml each) collected in heparin tubes (Becton, Dickinson and Company, USA) and purified according to the manufacturer’s recommendations for Lymphosep (Biowest, USA). The PBMCs were then stored in a -80°C deep freezer.

The study was approved by the Institutional Review Board of Asan Medical Center (2019–1676), adhered to the guidelines of the declaration of Helsinki. All the subjects or their legal guardians were informed about the study and they provided written informed consent. No potentially identifiable human data is presented in this study.

### Genotyping and imputation

Korean asthma patients were genotyped using the Korea Biobank Array, which was designed by the Korea National Institute of Health (KNIH), and based on the UK Biobank Axiom Array [28]. Low-quality SNPs were removed based on the following exclusion criteria: (1) poor clustering in the SNPolisher analysis, (2) genotype call rates < 95%, and (3) Hardy–Weinberg equilibrium *P*-value (HWE *P*) < 1 × 10^–6^. The genotype call rates and HWE *P*-values were calculated using PLINK v.1.9.0 [29]. And we excluded samples based on the following exclusion criteria: (1) low genotype call rates (< 95%), (2) deviating ± 3 standard deviation (SD) from the mean heterozygosity rate, (3) gender discrepancy, and (4) cryptic relatedness. All sample quality control (QC) process were performed using PLINK v.1.9.0, and three individuals were filtered. After QC was conducted, SNP imputation was performed using the Michigan imputation server [30]. Phasing and genotype imputations were performed using Eagle2 [31] and Minimac4 [32], respectively, and the ‘non-European’ or ‘mixed’ populations of the Haplotype Reference Consortium (HRC) were used as the reference panel [33]. We filtered out the low imputation quality SNPs, from the imputed SNPs, as follows: (1) low imputation quality score (Rsq < 0.3), (2) duplicated SNP, (3) HWE *P* < 1 × 10^–6^, (4) genotype call rates < 95%, and (5) minor allele frequency (MAF) < 1%. Finally, 6,676,632 SNPs for 433 samples were used further analysis.

### Transcriptome data processing

Total RNA sequencing was performed using the Swift RNA Library Kit and IDT Exome v2.0, according to the manufacturer’s instructions. Total RNA purity was determined by assaying 1 µL of the total RNA extract on a NanoDrop8000 spectrophotometer, and the integrity was checked using an Agilent Technologies 2100 Bioanalyzer, with an RNA integrity number value and the percentage of RNA fragments > 200 nt fragment distribution value. RNA sequencing was performed using an Illumina NovaSeq 6000 system, according to the protocols provided for 2 × 100 bp read sequencing. Quality trimming and adapter clipping of the reads were conducted using the Trimmomatic software [34]. Read alignment and quantification were performed using HISAT2 [35], SAM tools [36] and HTseq [37] with the Ensembl transcriptome reference (hg19/GRCh37).

### Normalization and quality control of the gene expressions

The gene expressions were quantified in transcripts per million (TPM) using GENCODE v19 [38]. A gene was allowed for downstream analyses if its expression in at least 20% of the samples was greater than 0.1 TPM and the read counts were greater than six. The expression values were normalized between the samples using the trimmed mean of *M*-values in edgeR [39], and each gene was normalized across the samples using inverse normal transformation.

### cis-eQTL analysis

To investigate the effects of genetic variation on gene expression in 433 Korean patients with asthma, we performed cis-eQTL analysis mapping using FastQTL [40]. After filtering out genes with low expression levels, we tested the association between normalized gene expression levels and genotypes of SNPs, adjusting for sex and 60 PEER factors according to the GTEx methods (https://gtexportal.org/home/methods) [41] as covariates. Only SNPs within a 1 Mb window from the transcription start site (TSS) of every gene were tested for cis-eQTL analysis.

FastQTL provides a permutation test to correct for multiple testing, therefore we performed an adaptive permutation scheme with the number of permutation iterations between 1,000 and 10,000, using the “–permute 1000 10,000” option. The beta distribution-adjusted empirical *P*-values were used to calculate false discovery rates (FDRs) using Storey’s method [42], and any gene with an FDR ≤ 5% was defined as an eGene whose expression was regulated by at least one SNP. To identify all the significant gene-SNP pairs associated with the expression of a particular gene, the nominal *P*-value thresholds of each eGene were defined as *P*_g, threshold_ = *F*_g,min_^−1^(*P*_t_), where *P*_t_ is the empirical *P*-value of the gene closest to the FDR of 5% and *F*^−1^ is the inverse cumulative distribution. All the SNPs with nominal *P*-values below the threshold for their target genes were defined as eSNPs that regulate the expression of certain genes.

### Comparison with the blood eQTL data of normal subjects

We compared our eQTL results against existing blood eQTL data from GTEx [43] and a Japanese study [44] to determine whether our eQTL signals overlapped with those of normal subjects or were asthma patient-specific. GTEx contains DNA and RNA-seq data from 838 individuals over 49 tissues and provides 8,663 eGenes and 1,052,542 cis-eQTLs for whole blood samples in GTEx v7. The Japanese study conducted an eQTL analysis on five immune cells and the whole blood from 105 healthy Japanese volunteers and provided 3,386 eGenes and 335,813 cis-eQTLs for whole blood. We identified the overlapping eGenes and compared the direction of the allelic effects of the overlapped cis-eQTLs between the eQTL data resources.

### Colocalization, SMR and TWAS analysis

To consider ethnic differences, we collected two asthma-GWAS summaries that originated from European (UK Biobank) [4] and Japanese ancestry [45] We then identified GWAS-lead SNPs in the genomic risk loci from each GWAS summary using the FUMA program, which provides functional annotation, visualization, and interpretation of GWAS results [46]. The GWAS-lead SNPs were clumped with following criteria: *P* < 5 × 10^–8^, distance between linkage disequilibrium (LD) blocks > 250 kb, and r^2^ < 0.1 from the 1,000 Genome Project phase 3 European panel [46]. Colocalization analyses between one of the three blood eQTL datasets (Korean asthma eQTL, GTEx, and Japanese) and the corresponding GWAS-lead SNPs were conducted using COLOC from the R package [15]. We selected target genes for the colocalization analyses with the following criteria: the TSS and the eQTL lead SNP of the gene were located within 0.5 Mb of the GWAS lead SNP in the GWAS risk locus. All the SNPs located between the GWAS lead SNP and the eQTL lead SNP or located in the 0.5 Mb extension on either side, were used for the analysis. We applied a posterior probability of colocalization (PP4) > 0.5 as the threshold for the colocalization significance. Significant GWAS-eQTL colocalization events were visualized using LocusCompareR in R [47].

We performed an SMR analysis with asthma-GWAS summary statistics and blood eQTLs using the SMR software [16]. The LD was estimated using a 1000 genome reference panel (European and East Asian) [48], and the major histocompatibility complex (MHC) region (chromosome 6:28.5 Mb ~ 33.5 Mb in hg19) was excluded from the analysis. We applied the Bonferroni correction and heterogeneity-independent instrument (HEIDI) threshold to exclude false positives and confounding effects.

In addition, we performed a TWAS analysis using the FUSION program [49]. Two inputs were required for FUSION program as follows: (1) GWAS summary statistics and (2) gene expression weights for each gene with significant heritability. We conducted the TWAS using two asthma-GWAS summaries from European and Japanese population, and two eQTL datasets, Korean asthmatic and GTEx eQTL data. The pre-computed gene expression weights for GTEx were provided in the FUSION program. For TWAS using Korean asthmatic data, we computed gene expression weight using individual genotype data and eQTL dataset, and succeeded in calculating gene expression weights for 1,160 genes that passed the gene expression heritability threshold (*P*_*h2*_ < 0.01). However, due to the unavailability of individual genotype data for Japanese eQTL, we did not perform TWAS for Japanese eQTL. We applied the Bonferroni correction to the results of TWAS (*P* < 4.31 × 10^–5^) and performed replication analysis using the GTEx dataset for significant TWAS genes.

### Druggable target genes

Using the drug-gene interaction database (DGIdb) [50], 15 asthma-related genes were mapped to licensed drugs to identify the druggable target genes. Information on drug indications for the target gene was obtained from CheMBL [51] and Drug Bank [52]. To identify the associations between the drugs and asthma, the identified drugs were queried on the Open Targets Platform [53]. We analyzed protein–protein interaction (PPI) between target genes using the STRING database [54].

## Results

### Cis-eQTL analysis of Korean asthma patients

A total of 433 Korean asthma patients were used for the eQTL analysis, and their basic characteristics are shown in Table 1. The mean age of these asthma patients, at diagnosis, was 45.3 ± 16.2 years, and 40.4% were male. Among them, 42.9% were current or ever smokers and 45.7% had atopy. The mean FEV1 and FEV1/forced vital capacity (FVC) were 75.2 ± 18.0% and 72.7 ± 13.4%, respectively. The mean blood eosinophil count and total IgE levels were 431.3 ± 448.6 (cells/µL) and 426.0 ± 686.7 IU/mL, respectively. Of the patients, 74.1% had blood eosinophils greater than or equal to 150 cells/µL, and 66.8% had total IgE greater than or equal to 100 IU/mL. Two hundred and twenty-five patients (53.8%) used a medium or high dose of inhaled corticosteroids (ICS).

**Table 1:** Characteristics of 433 Korean asthma patients

Characteristics	Korean asthma patients mean ± SD / N (%)
Age, years	49.5 ± 15.6
Sex (%)	
Male	175 (40.4)
Age at diagnosis, years^a^	45.3 ± 16.2
Age group at diagnosis^a^	
0 ~ 19	23
≥ 20	338
Smoking status (%)	
Current	53 (12.2)
Ever	133 (30.7)
Never	247 (57.0)
Atopy (%)	198 (45.7)
FEV1 pred.^a^ (%)	75.4 ± 18.0
< 80	223 (53.7)
≥ 80	192 (46.3)
FEV1/FVC^a^ (%)	72.8 ± 13.4
< 70	166 (40.0)
≥ 70	249 (60.0)
Blood eosinophil count^a^ (cells/µL)	431.3 ± 448.6
< 150	110 (25.9)
≥ 150	314 (74.1)
Dose of ICS^a^	
Medium or high dose	225 (53.8)
Low dose	193 (46.2)
Total IgE^a^ (IU/mL)	426.0 ± 686.7
< 100	128 (33.2)
≥ 100	257 (66.8)

All data are presented as the mean ± standard deviation (SD) or the number of participants (%)

^a^participants available with this data were analyzed

*FEV1 pred* predicted forced expiratory volume in 1 s, *FVC* Forced vital capacity, *ICS* Inhaled corticosteroids

**Fig. 1:**
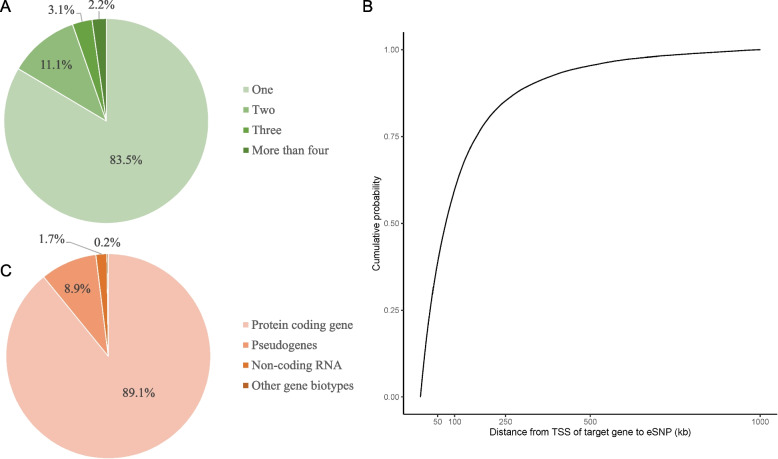
Characteristics of Korean asthmatic blood cis-eQTL. **A** Distribution of the target genes for each eSNP. **B** Cumulative distribution of the distance from the transcription start site (TSS) of the target gene to the eSNP. The x-axis is the distance from the TSS to the position of the eSNP, and the y-axis is the cumulative probability of the eSNP. **C** Distribution of the eGene biotypes. The biotypes were classified into four groups: protein-coding genes, pseudogenes, non-coding RNA, and other gene biotypes

We performed the cis-eQTL analysis with RNA-seq of PBMCs from the asthma patients using FastQTL [40]. Following QC for genotype and gene expressions, 6,676,632 SNPs and 24,066 genes were chosen for the analysis. After applying FDR < 0.05 as an eQTL *P*-value threshold, 267,691 SNPs (eSNPs) had at least one target gene (eGene), and 2,875 genes (eGenes) had at least one expression-associated SNP (eSNP); consequently 340,274 eGene-eSNP pairs (cis-eQTLs) were discovered (Additional File 1: Table S1). Of the 267,691 eSNPs, 223,578 (83.5%) had one target gene, 29,794 (11.1%) had two target genes, 8,346 (3.1%) had three target genes, and 5,974 (2.2%) had more than four target genes (Fig. 1A). Among the 340,274 eGene-eSNP pairs, the distance from the TSS of the target gene to the eSNP was within 500 kb in 95.4% (324,541 pairs), within 250 kb in 85.3% (290,253 pairs), within 100 kb in 60.0% (204,174 pairs), and within 50 kb in 39.0% (132,669 pairs) (Fig. 1B). The 2,875 eGenes were composed of 2,562 protein-coding genes (89.1%), 257 pseudogenes (8.9%), 50 non-coding RNAs (1.7%), and 5 other types of genes (0.2%) (Fig. 1C).

**Fig. 2:**
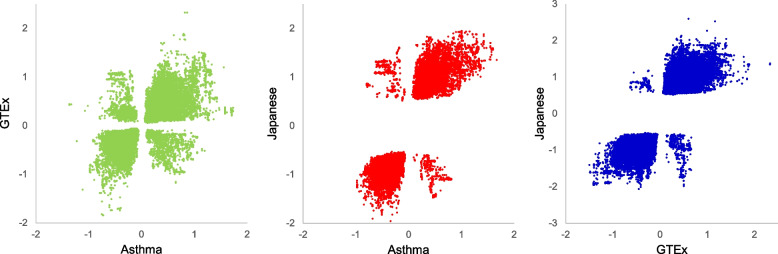
Scatter plots for comparisons of the direction of the allelic effects across the eQTL data. Scatter plots for comparisons of the direction of the allelic effects among the whole blood cis-eQTL data from the Korean asthma, GTEx, and Japanese populations. Each point in the scatter plot represents the allelic effect of an SNP on a gene expression. The scatter plots included 98,302 eQTLs between Korean asthma and GTEx, 71,533 eQTLs between Korean asthma and Japanese asthma, and 142,923 eQTLs between GTEx and Japanese asthma

We compared our blood eQTL results from asthmatic patients with healthy subjects. For this comparison, we used two blood eQTL datasets from a GTEx v7 study [43] and a Japanese study [44]. The total numbers of eGenes from the GTEx and Japanese datasets were 8,663 and 3,386, respectively. Among the 2,875 eGenes from the Korean asthmatic eQTL, 64.1% (1,844 of 2,875 eGenes) overlapped with the GTEx eGenes and 39.0% (1,122 of 2,875 eGenes) overlapped with the Japanese eGenes. For reference, 69.6% of the eGenes from the Japanese data (2,355/3,386) overlapped with the GTEx eQTL data. We also compared the direction of eSNP allelic effects on the target gene expressions between the asthmatic and normal eQTL data. There were 1,052,542 eGene-eSNP pairs in the GTEx and 335,813 eGene-eSNP pairs in the Japanese population. Among the 340,274 eGene-eSNP pairs from the Korean asthmatic eQTL data, 98,302 eGene-eSNP pairs were shared with GTEx and 71,533 eGene-eSNP pairs were shared with the Japanese pairs. Among the shared eGene-eSNP pairs, approximately 93.1% (91,508/98,302 pairs) for GTEx and 97.5% (69,758/71,533 pairs) for Japanese showed the same direction of allelic effects on gene expression as the Korean asthmatic eQTL (Fig. 2). As a reference, 142,923 eGene-eSNP pairs were shared between the GTEx and Japanese eQTL data, and 99.2% (141,842 pairs) showed allelic effects in the same direction (Fig. 2). As shown in Additional File 2: Table S2, we also found eQTL results showing allelic effects in the opposite direction, between the asthmatic and normal eQTL data as follows: 6.9% (6,794 pairs from 155 eGenes) of 98,302 pairs for GTEx, and 2.5% (1,775 pairs from 20 eGenes) of 71,533 pairs for the Japanese eQTL. Among the 155 eGenes from the GTEx data and 20 eGenes from the Japanese data, showing the opposite direction of the eQTL effect, 15 genes, including *CEACAM21*, *NDE1*, *LGALS8*, and AC124944.3, overlapped between them (Additional File 2: Table S2).

### Identification of the asthma-susceptibility genes from the Korean asthmatic blood eQTL data using COLOC, SMR and TWAS analysis

We performed a colocalization analysis to identify the target genes corresponding to the genetic variants from the asthma GWAS using the asthmatic eQTL data. We applied the COLOC method [15] to the three blood cis-eQTL datasets (Korean asthma, GTEx, and Japanese) and two asthma-GWAS summary statistics using European participants [4] and East Asian ancestries [45]. The European asthma-GWAS analyzed 64,538 asthma cases and 239,321 controls from the UK Biobank and identified 148 lead SNPs [4], whereas the Japanese asthma-GWAS analyzed 8,216 cases and 201,592 controls from a Japanese cohort and identified 11 lead SNPs [45] (Additional File 2: Table S3 and S4). We conducted the colocalization analysis by applying the criteria described in the Methods section and defined the GWAS colocalized genes with a PP4 greater than 0.5.

**Fig. 3:**
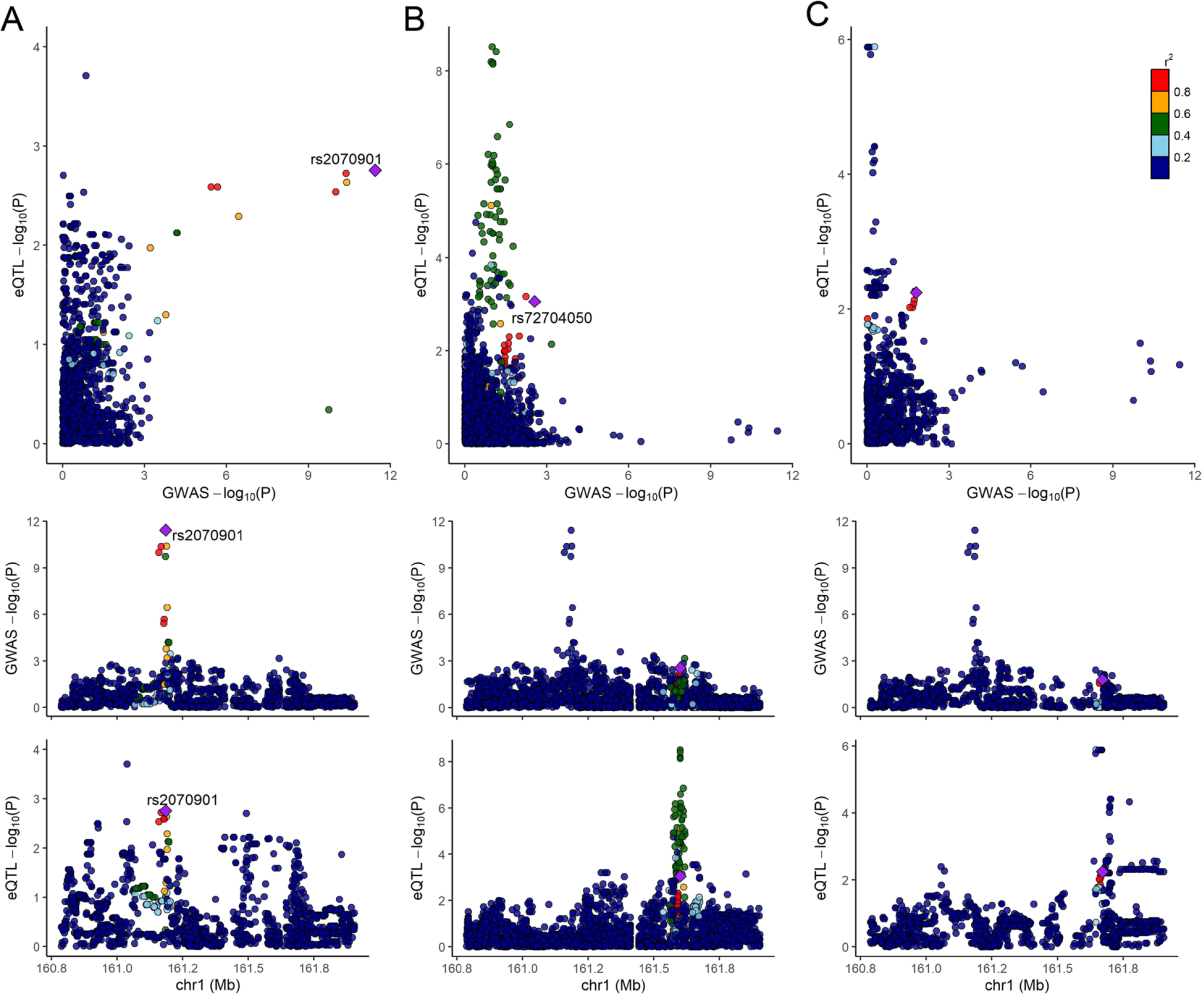
*FCGR3A* colocalization across the three eQTL data resources. Among all three eQTL datasets, namely the Korean asthmatic, GTEx, and Japanese eQTL data, *FCRG3A* showed a clear colocalization pattern in the Korean asthmatic data. The top panel presents a scatter plot to visualize the colocalization events, with eQTL *P*-values (y-axis) and GWAS *P*-values (x-axis). A bona fide signal should be present toward the top-right corner. The middle and bottom panels present regional plots for GWAS and eQTL *P*-values, respectively. **A** Korean asthmatic eQTL; **B** GTEx eQTL; and **C** Japanese eQTL

We identified 39 genes in the Korean asthmatic eQTL data with evidence of colocalization between the eQTL and European asthma-GWAS signals (36 genes) or Japanese asthma-GWAS signals (4 genes) (Additional File 2: Table S5). Among 39 genes, SUOX gene were identified both in European and Japanese asthma-GWASs. In addition, 59 and 39 genes were identified as having significant colocalization, from the normal eQTLs of GTEx and the Japanese study, respectively, using both the European and Japanese asthma-GWAS summaries (Additional File 2: Table S5). Of the 39 colocalized genes identified in the Korean asthmatic eQTL data, 20 genes were also significant in the colocalization results of the GTEx or Japanese eQTL datasets, 15 genes were not available in the GTEx or Japanese eQTL datasets, and the remaining 9 genes were found to be significant only in the Korean asthmatic eQTL dataset (Table 2, Fig. 3, and Additional File 3: Fig. S1-S9). For example, an association signal around rs2070901 located on chromosome 1 showed a clear colocalization pattern with the *FCRG3A* gene only in the Korean asthmatic eQTL data (Fig. 3). In the normal eQTL data of GTEx and the Japanese study, strong eQTL lead variants were identified in the LD block, different from that of the asthmatic eQTL data, with modest GWAS *P*-values. As a reference, the colocalization results for GTEx and the Japanese study showed 17 overlapping genes (Additional File 2: Table S6). Among these 17 overlapping genes, 10 were also colocalized in the Korean asthmatic eQTL data. Among the remaining seven genes, four were not available, and three did not show significant colocalization in the Korean asthmatic eQTL data.

**Table 2:** Korean asthma-specific colocalization genes

Study ID	Ensemble ID	Gene symbol	Var num	PP4 _Korean asthma_	PP4 _GTEx_	PP4 _Japanese_
GCST010042	ENSG00000124920	*MYRF*	796	0.890	3.25 × 10^–10^	0.304
GCST010042	ENSG00000185800	*DMWD*	1181	0.817	0.114	0.109
GCST010042	ENSG00000186088	*GSAP*	1097	0.979	0.002	0.096
GCST010042	ENSG00000173917	*HOXB2*	961	0.927	0.116	0.074
GCST010042	ENSG00000104147	*OIP5*	991	0.755	0.211	0.069
GCST010042	ENSG00000163608	*NEPRO*	1249	0.654	3.49 × 10^–6^	0.063
GCST010042	ENSG00000203747	*FCGR3A*	1281	0.619	9.39 × 10^–6^	0.053
GCST010042	ENSG00000197070	*ARRDC1*	711	0.982	2.90 × 10^–5^	0.016
GCST010042	ENSG00000260261	*AC124944.3*	1163	0.994	0.143	6.58 × 10^–4^

*Study ID* ID of the paper which provided asthma-GWAS summary, *Var num* number of genetic variants used in the colocalization analysis, *PP4* Posterior probability of colocalization, *NA* Not available because gene was out of analysis criteria or does not exist in the dataset

To identify asthma-associated genes using the asthmatic eQTL data, we additionally performed an SMR analysis using the asthmatic eQTL data and two asthma-GWAS summaries from Europe and Japan. SMR is generally used to discover genes satisfying the hypothesis that genetic variants affecting the risk of disease may act via changes in the expression levels of certain genes [16]. We applied the Bonferroni correction and HEIDI method for the SMR analysis, to reduce the likelihood of false-positive results.

After applying the Bonferroni correction (*P*_smr_ < 2.08 × 10^–6^) and HEIDI threshold (*P*_HEIDI_ > 0.01), a total of four SMR genes were identified: three SMR genes (*AHI*, *MED24*, and *DDX5*) with the European asthma-GWAS summary and one SMR gene (*RPS26*) with the Japanese asthma-GWAS summary (Additional File 2: Table S7). We also performed an SMR analysis using the GTEx and Japanese eQTL datasets by applying the Bonferroni correction (*P*_smr_ < 2.57 × 10^–6^ for GTEx and < 2.27 × 10^–6^ for Japanese) and HEIDI threshold (*P*_HEIDI_ > 0.01), and found 18 and one SMR genes, respectively (Additional File 2: Table S7).

Among the four SMR genes in the asthmatic eQTL data, two genes, *DDX5* and *MED24*, were not identified in the SMR analysis of the two normal eQTL data, and have not been reported to be significantly associated with asthma in the SMR analysis. In addition, *DDX5* were significant in both the colocalization and SMR analyses (Additional File 2: Table S5 and S7).

As another approach to identifying asthma-associated genes using the asthmatic eQTL data, we performed a TWAS analysis using the FUSION program [49]. TWAS is a gene-based association approach that investigates associations between genetically regulated gene expression and phenotype [17]. We carried out the TWAS analysis using two asthma-GWAS summaries from European and Japanese, and two eQTL datasets, Korean asthmatic and GTEx eQTL data. We tested 1,160 and 8,799 genes that passed the gene expression heritability threshold (*P*_h2_ < 0.01) for Korean asthma and GTEx, respectively.

After applying the Bonferroni correction to the TWAS analysis using Korean asthmatic eQTL dataset, we identified 22 significant genes (*P* < 4.31 × 10^–5^). Of these 22 genes, 20 and 3 genes identified from the TWAS results using European and Japanese GWASs, respectively (Additional File 2: Table S8). In addition, the *RPS26* gene was found to be significant in both European and Japanese asthma-GWASs. We then performed a replication analysis using GTEx data. Of the 22 TWAS genes, 6 were not available in the GTEx eQTL datasets. Among the remaining 16 genes, 11 were replicated in the GTEx TWAS analysis (*P* < 3.13 × 10^–3^ = 0.05/16), while five genes, including *INPP5B*, *LCLAT1*, *MYRF*, *TUBG2* and *CASTOR3*, were significant only in the Korean asthmatic dataset. Notably, the *MYRF* gene was also identified in colocalization analysis (Additional File 2: Table S5 and S8).

### Asthma-related genes specific to the Korean asthmatic blood eQTL data as therapeutic targets

In this study, using the asthmatic blood eQTL, we identified 15 asthma-related genes; two from the SMR, four from the TWAS, eight from the colocalization analysis, and one from both TWAS and colocalization analysis. To investigate the druggable genes, we mapped the 15 asthma-related genes onto licensed drugs or compounds from the DGIdb [50]. Of all the asthma-related genes, only *FCGR3A* (Fc gamma receptor IIIa) was found to interact with 24 drugs. We again investigated the 24 drugs in Drug Bank [52] and CheMBL [51], and confirmed that *FCGR3A* was associated with six asthma drugs that were approved or under clinical trials (Additional File 2: Table S9). Among these drugs, prednisolone, an approved asthma drug that targets the *NR3C1*, gene was found to interact with *FCGR3A*. We analyzed *FCGR3A*-*NR3C1* interaction using STRING, a database of known and predicted protein–protein interactions. While no direct interaction was observed between *FCGR3A* and *NR3C1*, we identified an interaction network comprising the path *FCGR3A*—*FCER1G*—*ZAP70*—*EGFR*—*NR3C1* based on known PPI results from curated databases and experiments. Additionally, three asthma drugs undergoing clinical trials were found to interact with *FCGR3A*, despite targeting *TNF* (adalimumab and etanercept) and *PTGS1*/*PTGS2* (indomethacin). No direct interaction was observed between *FCGR3A* and *TNF* or between *FCGR3A* and *PTGS1*/*PTGS2*. However, similar to the PPI result of *FCGR3A* and *NR3C1*, we identified an interaction network comprising the path *FCGR3A*—*FCER1G*—*ZAP70*—*TNF*. Furthermore, we found an interaction network comprising the path *FCGR3A*—*SRC*—*PTGS1*/*PTGS2* based on the known PPI results from curated databases and experiment.

## Discussion

The main aim of this study was to identify asthma-related genes from asthmatic eQTL data, given that gene expression may change in a context-specific manner depending on the disease status. We identified 15 asthma-related genes specific to the asthmatic eQTL data, by three methods of colocalization, SMR and TWAS, using two asthma-GWAS summary statistics from European and Japanese populations, and suggested these genes as therapeutic targets for asthma.

Among the 9 colocalization genes specific to Korean asthma, *FCGR3A* is well known for its association with various innate immune responses [55], and has been reported to interact with certain approved asthma drugs (Additional File 2: Table S9). As shown in Fig. 3, *FCGR3A* was highly colocalized with the eQTL and GWAS variants only in the Korean asthmatic eQTL data. In the normal blood eQTL datasets, eQTL variants around the GWAS-lead SNP rs2070901 were not found to be significant. The eQTL *P*-values of rs2070901 were 0.0014, 0.53, and 0.07 in the Korean, GTEx, and Japanese, respectively. Additionally, *ARRDC1* (arrestin domain containing 1) is located in the extracellular vesicles (EVs), which are known to contribute to the pathogenesis of asthma via various mechanisms related to both inflammation and pathological remodeling [56].

Interestingly, among the colocalization genes, four genes (*MYRF*, *DMWD*, *GSAP*, and *NEPRO*) were involved in neuronal survival and proliferation. *MYRF* (myelin regulatory factor) encodes a transcription factor required for myelination of the central nervous system. This gene is located near the fatty acid desaturase (FADS) gene cluster and is associated with an increased risk of inflammation [57]. *DMWD* (DM1 locus, WD repeat containing) is predicted to be located in the dendrites, nuclei, and perikarya of neurons, however its function is poorly characterized. The amino acid sequence of *DMWD* is similar to that of WD repeat domain 20 (*WDR20*), which is associated with childhood asthma [58]. In addition, *GSAP* (gamma-secretase activating protein) plays an important role in Alzheimer's disease by regulating lipid homeostasis and mitochondrial function [59], but its functional relevance in asthma is not largely unknown. *NEPRO* (nucleolus and neural progenitor protein) is predicted to be located in the nucleolus and is known to act as a Notch effector for the development and maintenance of neural progenitor cells in the neocortex [60]. We do not know how to link the functions of these genes to asthma, and future studies are required to determine their underlying mechanisms. The remaining colocalization genes belong to embryo development (*HOXB2*) [61], cell division (*OIP5*) [62], and one with an unknown function (*AC124944.3*).

Among the two asthma-related genes identified through the SMR analysis, *MED24* is associated with increased concentrations of phosphatidylcholine, a metabolite that is positively associated with asthma [63]. In addition, *DDX5* is known to be a hub gene of the cilia module in asthma and a regulator of *ERBB2,* which is associated with epithelial repair processes in asthma patients [64, 65].

Through a TWAS analysis, five genes related to asthma were identified, including the *MYRF* gene which was also identified in colocalization analysis. The *INPP5B* gene encodes an inositol polyphosphate-5-phosphatase B protein that regulates calcium signaling, which is involved in inflammatory cellular responses and interactive signaling pathways that mediate the development of asthma [66]. The *LCLAT1* gene encodes a lysocardiolipin acyltransferase 1 protein that is involved in phosphatidylinositol acyl-chain remodeling. Researchers have found that changes in microRNA (miRNA) expression can contribute to the pathogenesis of asthma, and there is a negative correlation between *LCLAT1* and miRNAs (miR-199b-5p and miR-223-3p) associated with asthma [67]. The *CASTOR3* gene regulates amino acid metabolism, which plays a role in various anti-oxidant and immunological activities relevant to asthma pathogenesis. And differences in amino acid levels may be involved in the development of asthma [68]. Additionally, the *TUBG2* gene encodes a tubulin gamma-2 chain protein, which is a major constituent of microtubules. Microtubules play a role in smooth muscle cell migration and airway remodeling a key feature of asthma [69].

This study had several limitations. First, our eQTL analysis was performed only in Korean asthma patients only, without comparable eQTL data from normal Koreans. Therefore, we compared our findings with two normal blood eQTL datasets of European and Japanese ancestry. Second, we used two asthma-GWAS summaries of European and Asian ancestry for the colocalization, SMR, and TWAS analysis, with different sample sizes (64,538 cases vs. 8,216 cases) for the asthma GWAS. Both of these limitations can affect the power of the analysis and contribute to bias related to ethnicity due to differences in LD structures between populations. To validate our findings, future analyses will require a comparison with normal Korean eQTL data and a larger sample size for the asthma-GWAS of East Asian ancestry. Third, through the drug target analysis, we discovered that the *FCGR3A* gene interacts with asthma drugs that have either been approved or are currently undergoing clinical trials. While we did not observe direct interactions between *FCGR3A* and drug target genes, we identified indirect interaction network based on the curated databases and experiments. These interactions could have potential implications for asthma research and treatment, but further investigation into these interactions could be necessary to fully understand their potential impact. Another limitation is that we used PBMCs, which may not fully capture the disease context. Using asthma-relevant tissues such as bronchial epithelial cells, airway smooth muscle cells, and nasal epithelial cells can provide more accurate insights into the molecular mechanisms of asthma and help identify eQTLs that are specific to these tissues. This limitation may affect our ability to identify eQTLs specific to asthma-relevant tissues and may impact the interpretation of our results, so our findings will need to be confirmed in additional asthma-relevant tissues.

## Conclusions

This study provided asthma cis-eQTL data and identified 15 asthma-related genes by integrating them with GWAS data. We envisage that our findings will contribute to understanding the etiological mechanisms of asthma and provide novel therapeutic targets.

### Supplementary Information

**Additional file 1: Table S1. **Results of cis-eQTLs in 433 Korean patients with asthma.

**Additional file 2: Table S2. **eQTL results showing allelic effects in the opposite direction, between the asthmatic and normal eQTL data; **Table S3.** Results for 148 loci significantly associated with asthma in the GWAS analysis of the UK Biobank for colocalization analysis; **Table S4.** Results for 11 loci significantly associated with asthma in the GWAS analysis of the Japanese cohort for colocalization analysis; **Table S5.** Significant colocalization genes of three eQTL datasets; **Table S6.** Shared eGenes of significant colocalization between GTEx and Japanese; **Table S7.** Significant results of SMR analysis; **Table S8.** Significant results of TWAS analysis; **Table S9**. Asthma susceptibility genes and interacting asthma drugs.

**Additional file 3: Fig. S1-S9.**

## Data Availability

The datasets supporting the conclusions of this article are included within the article and its additional files. And the RNA-seq data discussed in this publication have been deposited in NCBI's Gene Expression Omnibus and are accessible through GEO Series accession number GSE226825 (https://www.ncbi.nlm.nih.gov/geo/query/acc.cgi?acc=GSE226825).

## Abbreviations

GWASs: Genome-wide association studies
SNPs: Single-nucleotide polymorphisms
eQTL: Expression quantitative trait loci
SMR: Summary-based Mendelian Randomization
TWAS: Transcriptome-wide association study
pQTL: Protein quantitative trait loci
GTEx: Genotype-Tissue Expression
PBMCs: Peripheral blood mononuclear cells
FEV1: Forced expiratory volume in 1 second
PC: Provocation concentration
KNIH: Korea National Institute of Health
HWE P: Hardy-Weinberg equilibrium P-value
SD: Standard deviation
QC: Quality control
HRC: Haplotype Reference Consortium
MAF: Minor allele frequency
TPM: Transcripts per million
TSS: Transcription start site
FDRs: False discovery rates
LD: Linkage disequilibrium
PP4: Posterior probability of colocalization
MHC: Major histocompatibility complex
HEIDI: Heterogeneity-independent instrument
DGIdb: Drug-gene interaction database
PPI: Protein-protein interaction
FVC: Forced vital capacity
ICS: Inhaled corticosteroids
CEACAM21: CEA cell adhesion molecule 21
NDE1: nudE neurodevelopment protein 1
LGALS8: Galectin 8
SUOX: Sulfite oxidase
FCGR3A: Fc gamma receptor IIIa
AHI: Abelson helper integration site 1
MED24: Mediator complex subunit 24
DDX5: DEAD-box helicase 5
RPS26: Ribosomal protein S26
INPP5B: Inositol polyphosphate-5-phosphatase B
LCLAT1: Lysocardiolipin acyltransferase 1
MYRF: Myelin regulatory factor
TUBG2: Tubulin gamma 2
CASTOR3: CASTOR family member 3
NR3C1: Nuclear receptor subfamily 3 group C member 1
FCER1G: Fc epsilon receptor Ig
ZAP70: Zeta chain of T cell receptor associated protein kinase 70
EGFR: Epidermal growth factor receptor
TNF: Tumor necrosis factor
PTGS1: Prostaglandin-endoperoxide synthase 1
PTGS2: Prostaglandin-endoperoxide synthase 2
SRC: SRC proto-oncogene, non-receptor tyrosine kinase
ARRDC1: Arrestin domain containing 1
EVs: Extracellular vesicles
FADS: Fatty acid desaturase
DMWD: DM1 locus, WD repeat containing
WDR20: WD repeat domain 20
GSAP: Gamma-secretase activating protein
NEPRO: Nucleolus and neural progenitor protein
HOXB2: Homeobox B2
OIP5: Opa interacting protein 5
ERBB2: Erb-b2 receptor tyrosine kinase 2
miRNA: MicroRNA
