# Supplementary material for: Identification of asthma-related genes using asthmatic blood eQTLs of Korean patients
Source: BMC Med Genomics. 2023 Oct 24;16:259. doi: 10.1186/s12920-023-01677-7 (PMC10599017; doi:10.1186/s12920-023-01677-7)
Supplement: Supplementary file 3 — Additional file 3: Fig. S1-S9. [file 12920_2023_1677_MOESM3_ESM.pdf]

MYRF

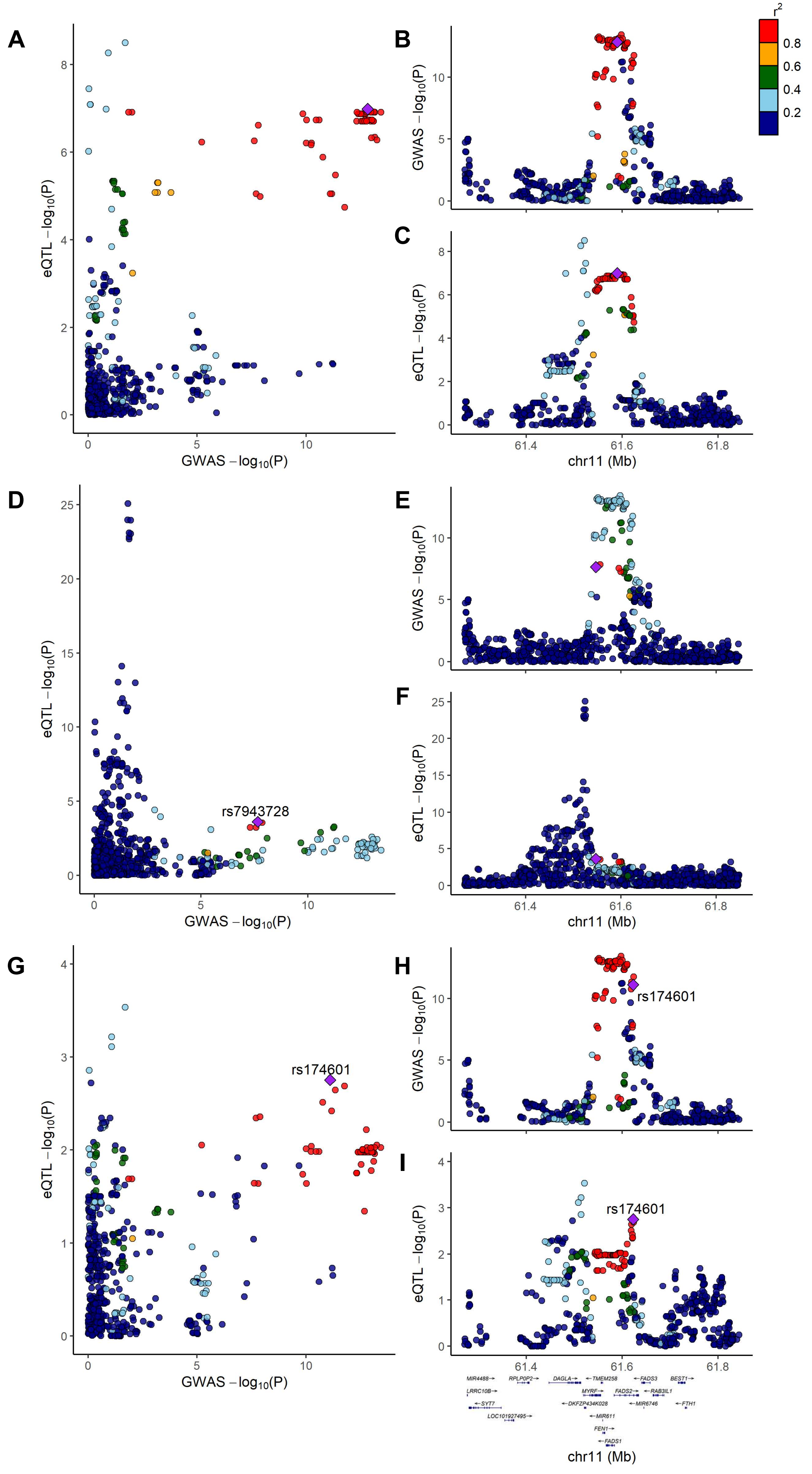

A
B
C
D
E
F
G
H
I
r2
0.8
0.6
0.4
0.2
eQTL −log10(P)
GWAS −log10(P)
chr11 (Mb)
rs7943728
rs174601
MIR4488
RPLP0P2
DAGLA
TMEM258
FADS3
BEST1
LRRC10B
MYRF
FADS2
RAB3IL1
SYT7
DKFZP434K028
MIR6746
FTH1
LOC101927495
MIR611
FEN1
FADS1

Fig. S1. *MYRF* colocalization across the three eQTL data resources.

A-C. Results of colocalization analysis using Korean asthmatic eQTL. **D-F.** Results of colocalization analysis using GTEx eQTL. **G-I.** Results of colocalization analysis using Japanese eQTL. **A, D, G** These panels present scatter plots to visualize the colocalization events, with eQTL *P*-values (y-axis) and GWAS *P*-values (x-axis). A bona fide signal should be present toward the top-right corner. **B, E, H** These panels present regional plots for GWAS *P*-values. The x-axis represents chromosomal base-pair position, and the y-axis represents $-\log_{10}(P\text{-value})$. **C, F, I** These panels present regional plots for eQTL *P*-values. The x-axis represents chromosomal base-pair position, and the y-axis represents $-\log_{10}(P\text{-value})$.

DMWD

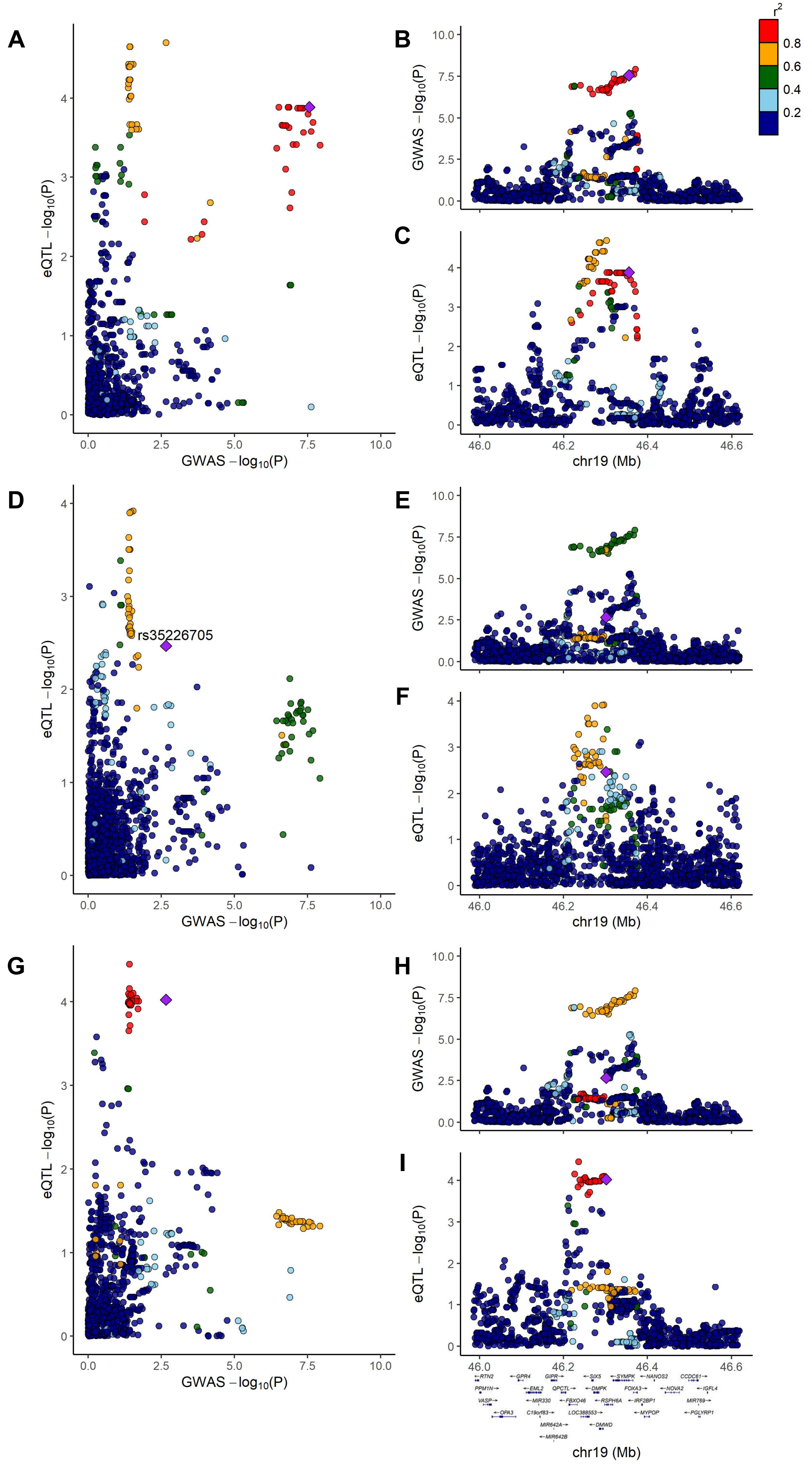
A
B
C
D
E
F
G
H
I
eQTL −log10(P)
GWAS −log10(P)
chr19 (Mb)
r2
0.8
0.6
0.4
0.2
rs35226705
←RTN2
←GPR4
GIPR→
←SIX5
←SYMPK
←NANOS2
CCDC61→
PPM1N→
←EML2
QPCTL→
←DMPK
FOXA3→
←NOVA2
←IGFL4
VASP→
←MIR330
←FBXO46
←RSPH6A
←IRF2BP1
MIR769→
←OPA3
C19orf83→
LOC388553→
←MYPOP
←PGLYRP1
MIR642A→
←DMWD
←MIR642B

Fig. S2. *DMWD* colocalization across the three eQTL data resources.

A-C. Results of colocalization analysis using Korean asthmatic eQTL. **D-F.** Results of colocalization analysis using GTEx eQTL. **G-I.** Results of colocalization analysis using Japanese eQTL. **A, D, G** These panels present scatter plots to visualize the colocalization events, with eQTL *P*-values (y-axis) and GWAS *P*-values (x-axis). A bona fide signal should be present toward the top-right corner. **B, E, H** These panels present regional plots for GWAS *P*-values. The x-axis represents chromosomal base-pair position, and the y-axis represents $-\log_{10}(P\text{-value})$. **C, F, I** These panels present regional plots for eQTL *P*-values. The x-axis represents chromosomal base-pair position, and the y-axis represents $-\log_{10}(P\text{-value})$.

GSAP

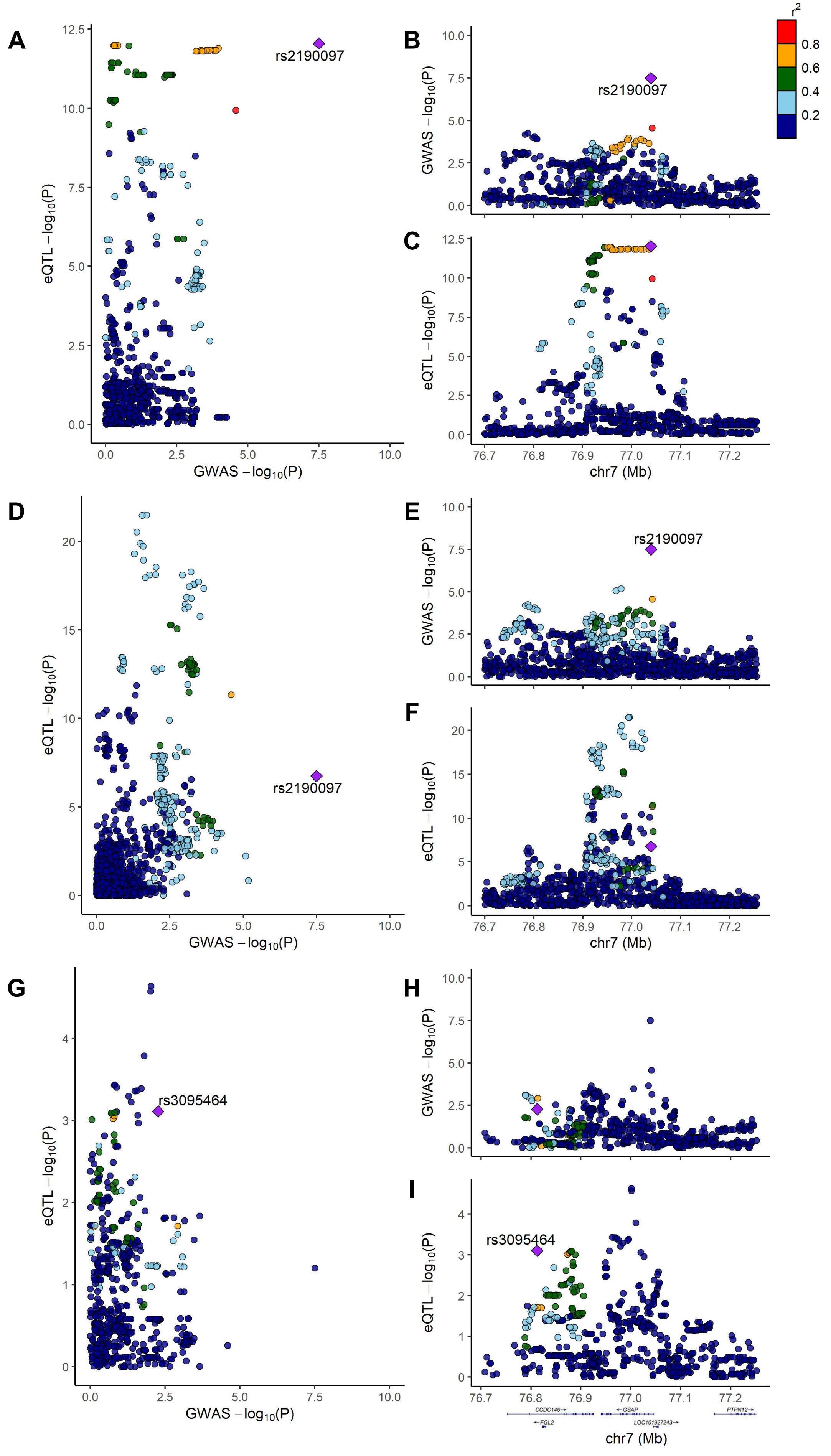
A
B
C
D
E
F
G
H
I
rs2190097
rs3095464
eQTL −log10(P)
GWAS −log10(P)
chr7 (Mb)
r2
0.8
0.6
0.4
0.2
CCDC146
GSAP
PTPN12
FGL2
LOC101927243

Fig. S3. *GSAP* colocalization across the three eQTL data resources.

A-C. Results of colocalization analysis using Korean asthmatic eQTL. **D-F.** Results of colocalization analysis using GTEx eQTL. **G-I.** Results of colocalization analysis using Japanese eQTL. **A, D, G** These panels present scatter plots to visualize the colocalization events, with eQTL *P*-values (y-axis) and GWAS *P*-values (x-axis). A bona fide signal should be present toward the top-right corner. **B, E, H** These panels present regional plots for GWAS *P*-values. The x-axis represents chromosomal base-pair position, and the y-axis represents $-\log_{10}(P\text{-value})$. **C, F, I** These panels present regional plots for eQTL *P*-values. The x-axis represents chromosomal base-pair position, and the y-axis represents $-\log_{10}(P\text{-value})$.

HOXB2

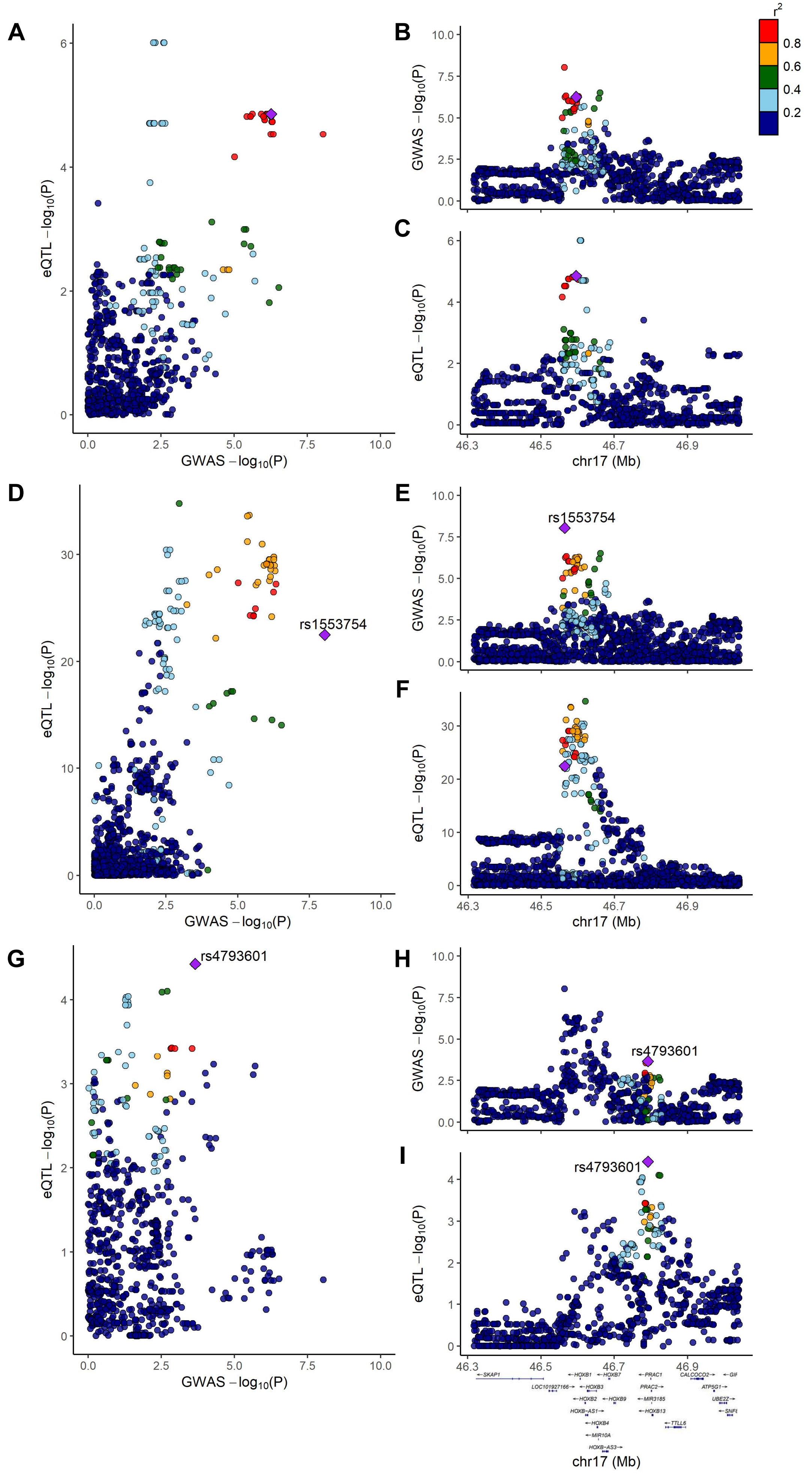

A
B
C
D
E
F
G
H
I
r2
0.8
0.6
0.4
0.2
eQTL −log10(P)
GWAS −log10(P)
chr17 (Mb)
rs1553754
rs4793601
SKAP1
HOXB1
HOXB7
PRAC1
CALCOCO2
LOC101927166
HOXB3
PRAC2
ATP5G1
HOXB2
HOXB9
MIR3185
UBE2Z
HOXB-AS1
HOXB13
HOXB4
TTLL6
MIR10A
HOXB-AS3

Fig. S4. *HOXB2* colocalization across the three eQTL data resources.

A-C. Results of colocalization analysis using Korean asthmatic eQTL. **D-F.** Results of colocalization analysis using GTEx eQTL. **G-I.** Results of colocalization analysis using Japanese eQTL. **A, D, G** These panels present scatter plots to visualize the colocalization events, with eQTL *P*-values (y-axis) and GWAS *P*-values (x-axis). A bona fide signal should be present toward the top-right corner. **B, E, H** These panels present regional plots for GWAS *P*-values. The x-axis represents chromosomal base-pair position, and the y-axis represents $-\log_{10}(P\text{-value})$. **C, F, I** These panels present regional plots for eQTL *P*-values. The x-axis represents chromosomal base-pair position, and the y-axis represents $-\log_{10}(P\text{-value})$.

OIP5

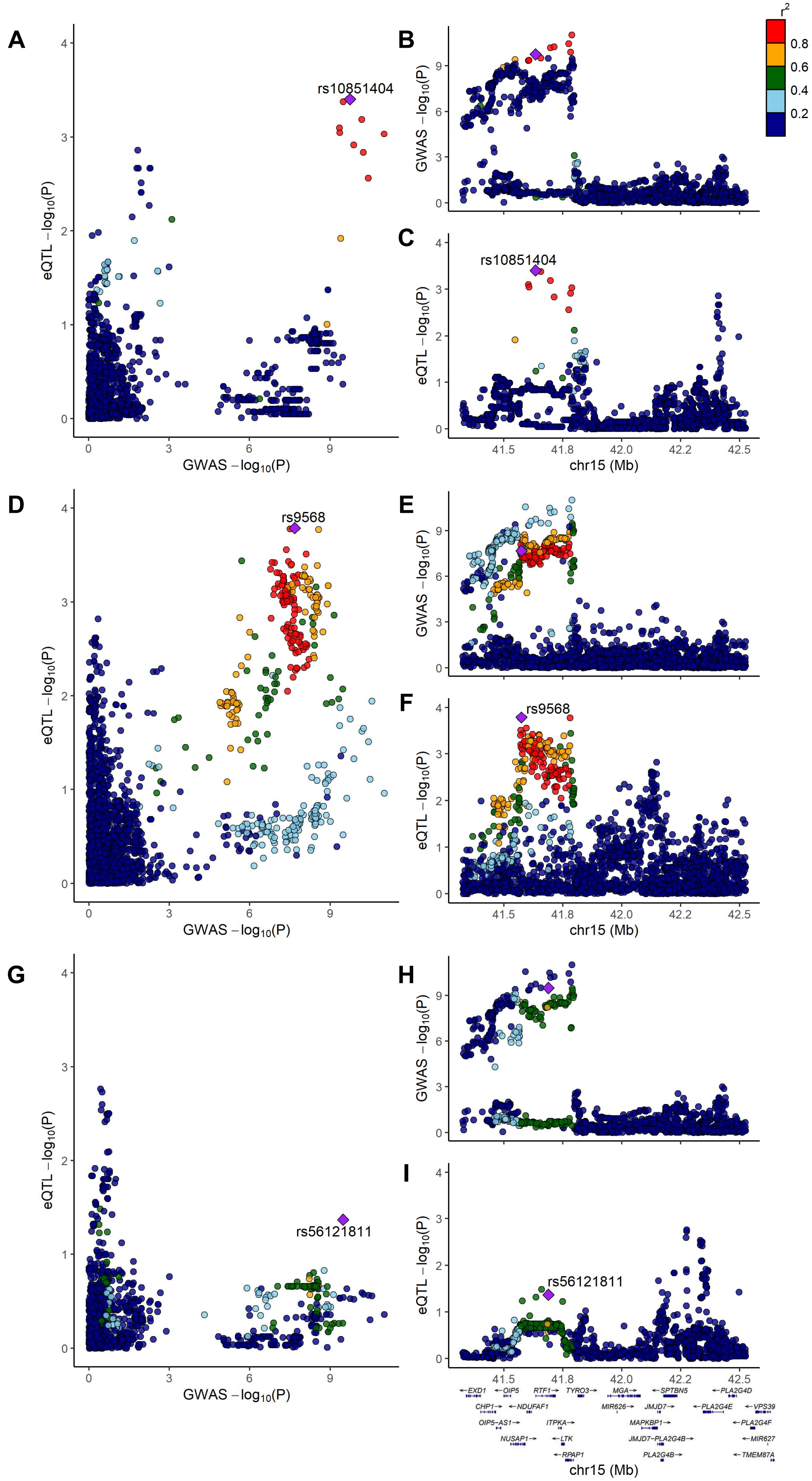
A
B
C
D
E
F
G
H
I
rs10851404
rs9568
rs56121811
eQTL −log10(P)
GWAS −log10(P)
chr15 (Mb)
r2
0.8
0.6
0.4
0.2
41.5
41.8
42.0
42.2
42.5
EXD1
OIP5
RTF1
TYRO3
MGA
SPTBN5
PLA2G4D
CHP1
NDUFAF1
MIR626
JMJD7
PLA2G4E
VPS39
OIP5−AS1
ITPKA
MAPKBP1
PLA2G4F
NUSAP1
LTK
JMJD7−PLA2G4B
MIR627
RPAP1
PLA2G4B
TMEM87A

Fig. S5. *OIP5* colocalization across the three eQTL data resources.

A-C. Results of colocalization analysis using Korean asthmatic eQTL. **D-F.** Results of colocalization analysis using GTEx eQTL. **G-I.** Results of colocalization analysis using Japanese eQTL. **A, D, G** These panels present scatter plots to visualize the colocalization events, with eQTL *P*-values (y-axis) and GWAS *P*-values (x-axis). A bona fide signal should be present toward the top-right corner. **B, E, H** These panels present regional plots for GWAS *P*-values. The x-axis represents chromosomal base-pair position, and the y-axis represents $-\log_{10}(P\text{-value})$. **C, F, I** These panels present regional plots for eQTL *P*-values. The x-axis represents chromosomal base-pair position, and the y-axis represents $-\log_{10}(P\text{-value})$.

NEPRO

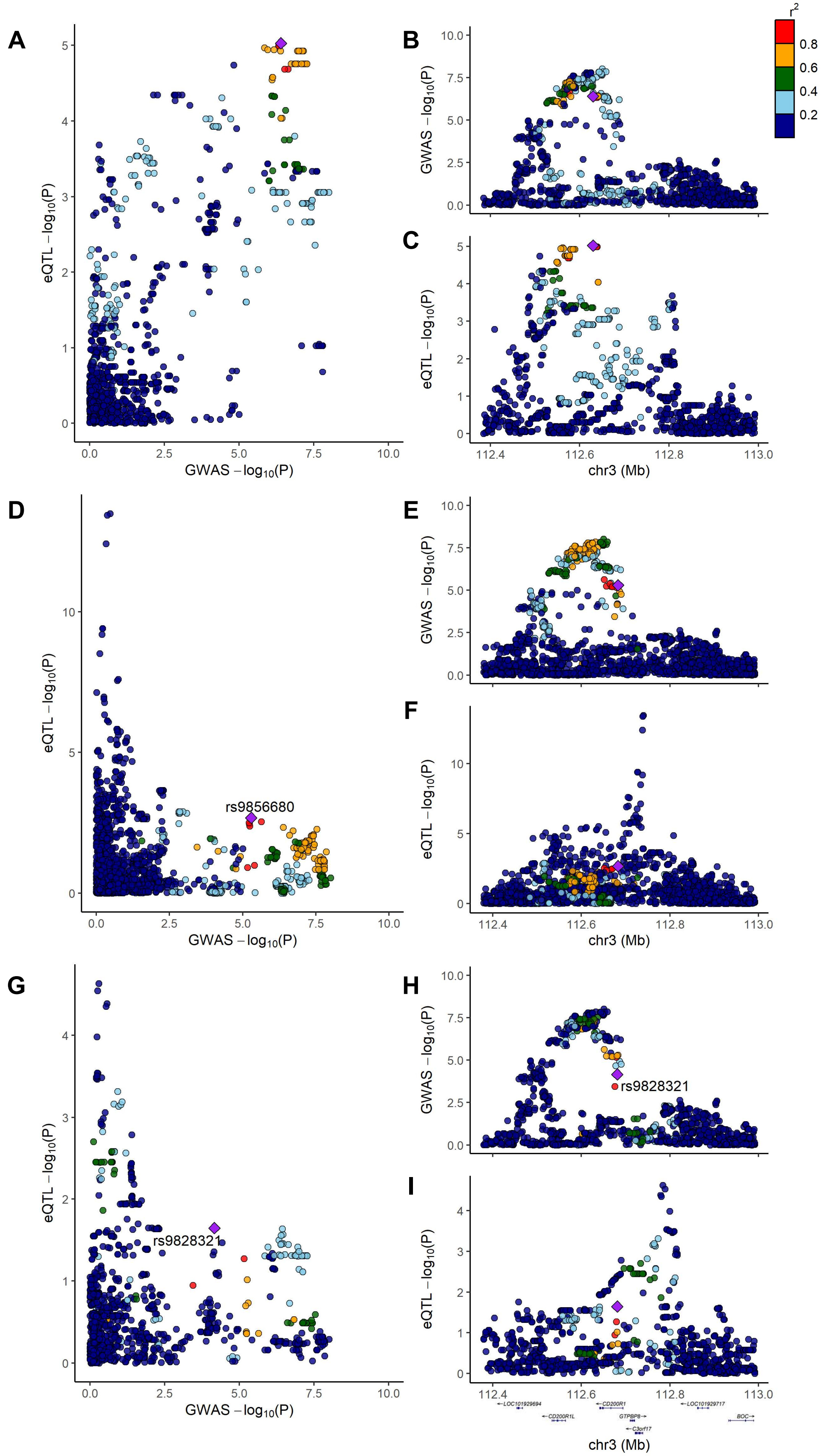
A
B
C
D
E
F
G
H
I
r²
0.8
0.6
0.4
0.2
eQTL −log10(P)
GWAS −log10(P)
chr3 (Mb)
rs9856680
rs9828321
LOC101929694
CD200R1L
CD200R1
GTPBP8
C3orf17
LOC101929717
BOC

Fig. S6. *NEPRO* colocalization across the three eQTL data resources.

A-C. Results of colocalization analysis using Korean asthmatic eQTL. **D-F.** Results of colocalization analysis using GTEx eQTL. **G-I.** Results of colocalization analysis using Japanese eQTL. **A, D, G** These panels present scatter plots to visualize the colocalization events, with eQTL *P*-values (y-axis) and GWAS *P*-values (x-axis). A bona fide signal should be present toward the top-right corner. **B, E, H** These panels present regional plots for GWAS *P*-values. The x-axis represents chromosomal base-pair position, and the y-axis represents $-\log_{10}(P\text{-value})$. **C, F, I** These panels present regional plots for eQTL *P*-values. The x-axis represents chromosomal base-pair position, and the y-axis represents $-\log_{10}(P\text{-value})$.

FCGR3A

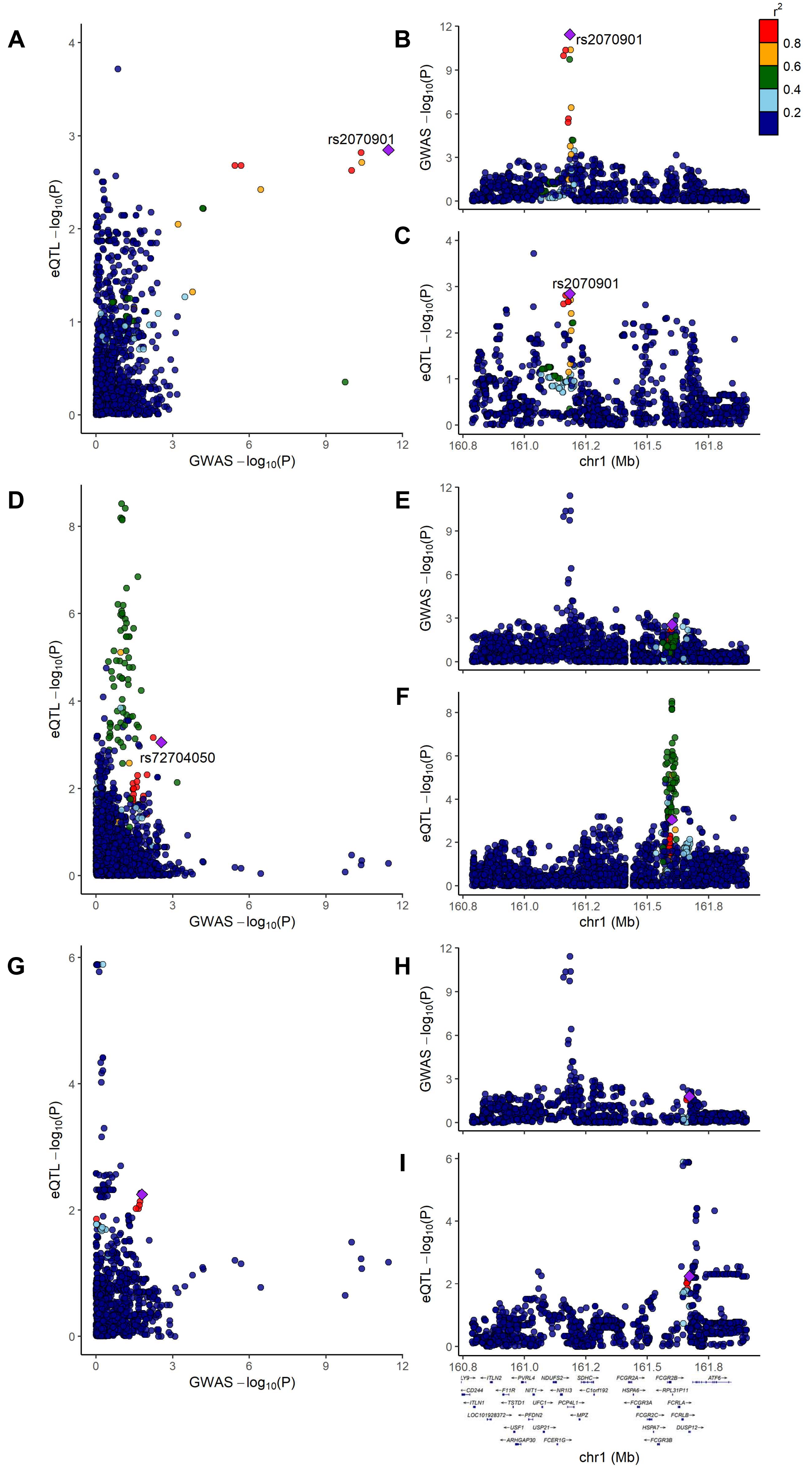
A
B
C
D
E
F
G
H
I
rs2070901
rs2070901
rs2070901
rs72704050
eQTL −log10(P)
GWAS −log10(P)
chr1 (Mb)
r2
0.8
0.6
0.4
0.2
LY9→ ←ITLN2 ←PVRL4 NDUFS2→ SDHC→ FCGR2A→ FCGR2B→ ATF6→
←CD244 ←F11R NIT1→ ←NR1I3 ←C1orf192 HSPA6→ ←RPL31P11
←ITLN1 ←TSTD1 UFC1→ PCP4L1→ ←FCGR3A FCRLA→
LOC101928372→ ←PFDN2 ←MPZ FCGR2C→ FCRLB→
←USF1 USP21→ HSPA7→ DUSP12→
←ARHGAP30 FCER1G→ ←FCGR3B

Fig. S7. *FCGR3A* colocalization across the three eQTL data resources.

A-C. Results of colocalization analysis using Korean asthmatic eQTL. **D-F.** Results of colocalization analysis using GTEx eQTL. **G-I.** Results of colocalization analysis using Japanese eQTL. **A, D, G** These panels present scatter plots to visualize the colocalization events, with eQTL *P*-values (y-axis) and GWAS *P*-values (x-axis). A bona fide signal should be present toward the top-right corner. **B, E, H** These panels present regional plots for GWAS *P*-values. The x-axis represents chromosomal base-pair position, and the y-axis represents $-\log_{10}(P\text{-value})$. **C, F, I** These panels present regional plots for eQTL *P*-values. The x-axis represents chromosomal base-pair position, and the y-axis represents $-\log_{10}(P\text{-value})$.

ARRDC1

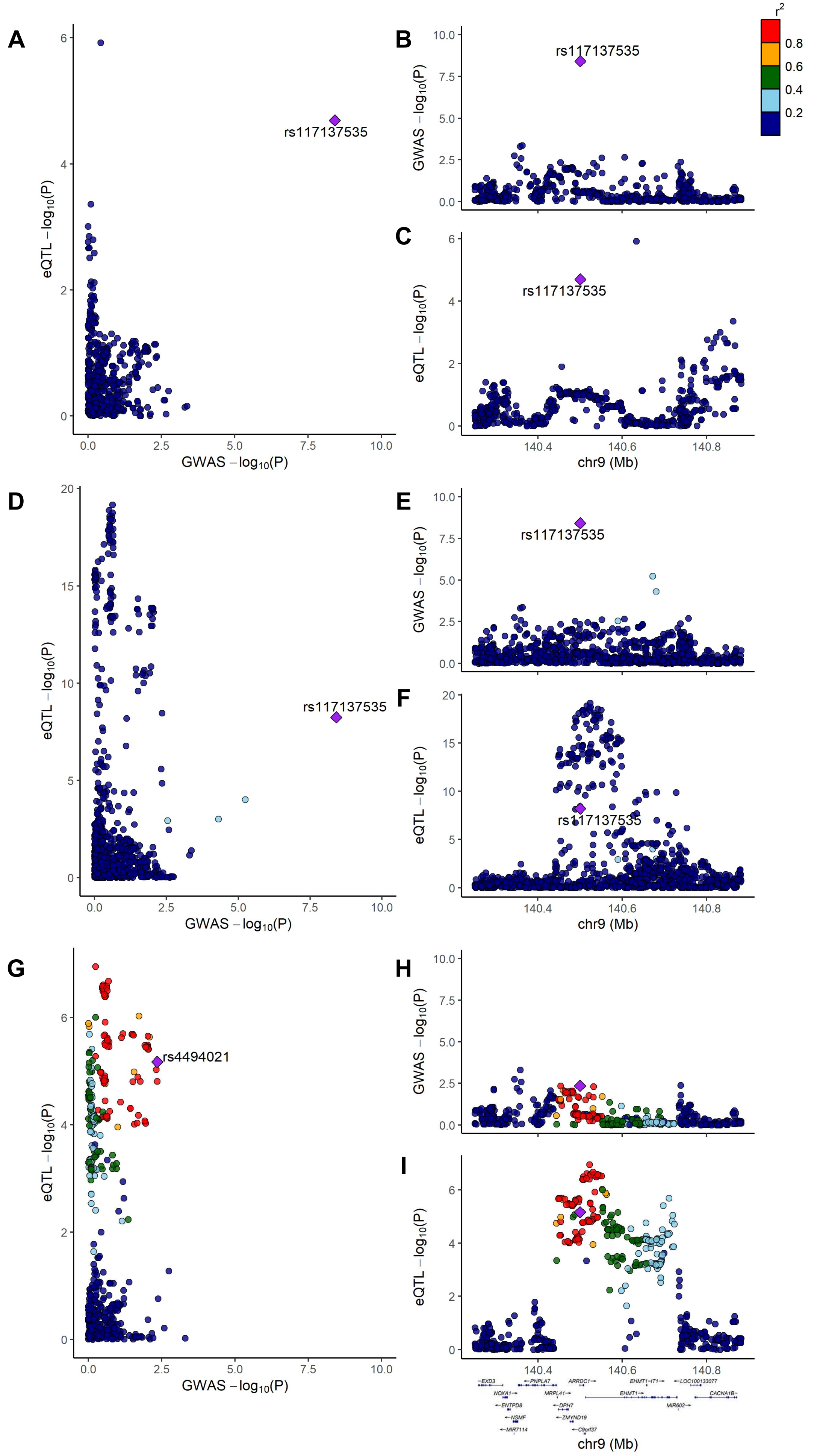

A
B
C
D
E
F
G
H
I
rs117137535
rs4494021
GWAS −log10(P)
eQTL −log10(P)
chr9 (Mb)
r2
0.8
0.6
0.4
0.2
EXD3
PNPLA7
ARRDC1
EHMT1-IT1
LOC100133077
NOXA1
MRPL41
EHMT1
CACNA1B
ENTPD8
DPH7
MIR602
NSMF
ZMYND19
MIR7114
C9orf37

Fig. S8. *ARRDC1* colocalization across the three eQTL data resources.

A-C. Results of colocalization analysis using Korean asthmatic eQTL. **D-F.** Results of colocalization analysis using GTEx eQTL. **G-I.** Results of colocalization analysis using Japanese eQTL. **A, D, G** These panels present scatter plots to visualize the colocalization events, with eQTL *P*-values (y-axis) and GWAS *P*-values (x-axis). A bona fide signal should be present toward the top-right corner. **B, E, H** These panels present regional plots for GWAS *P*-values. The x-axis represents chromosomal base-pair position, and the y-axis represents $-\log_{10}(P\text{-value})$. **C, F, I** These panels present regional plots for eQTL *P*-values. The x-axis represents chromosomal base-pair position, and the y-axis represents $-\log_{10}(P\text{-value})$.

AC124944.3

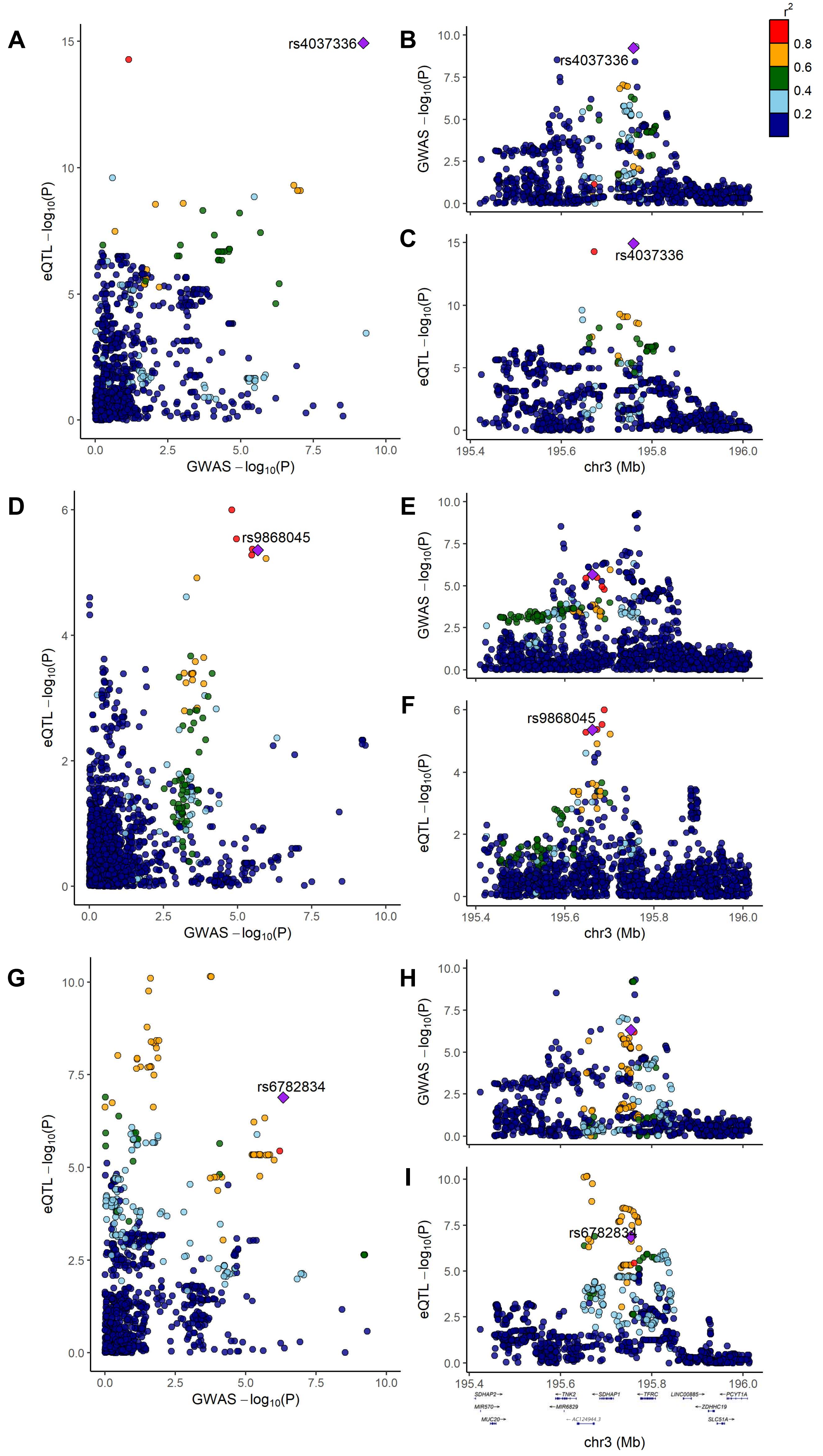

A
rs4037336
eQTL −log10(P)
GWAS −log10(P)
B
rs4037336
GWAS −log10(P)
C
rs4037336
eQTL −log10(P)
chr3 (Mb)
r2
0.8
0.6
0.4
0.2
D
rs9868045
eQTL −log10(P)
GWAS −log10(P)
E
GWAS −log10(P)
F
rs9868045
eQTL −log10(P)
chr3 (Mb)
G
rs6782834
eQTL −log10(P)
GWAS −log10(P)
H
GWAS −log10(P)
I
rs6782834
eQTL −log10(P)
SDHAP2
TNK2
SDHAP1
TFRC
LINC00885
PCYT1A
MIR570
MIR6829
ZDHHC19
MUC20
AC124944.3
SLC51A
chr3 (Mb)

Fig. S9. *AC124944.3* colocalization across the three eQTL data resources.

A-C. Results of colocalization analysis using Korean asthmatic eQTL. **D-F.** Results of colocalization analysis using GTEx eQTL. **G-I.** Results of colocalization analysis using Japanese eQTL. **A, D, G** These panels present scatter plots to visualize the colocalization events, with eQTL *P*-values (y-axis) and GWAS *P*-values (x-axis). A bona fide signal should be present toward the top-right corner. **B, E, H** These panels present regional plots for GWAS *P*-values. The x-axis represents chromosomal base-pair position, and the y-axis represents $-\log_{10}(P\text{-value})$. **C, F, I** These panels present regional plots for eQTL *P*-values. The x-axis represents chromosomal base-pair position, and the y-axis represents $-\log_{10}(P\text{-value})$.
